# Veno-Arterial Extracorporeal Membrane Oxygenation (VA-ECMO) Support in New Era of Heart Transplant

**DOI:** 10.3389/ti.2024.12981

**Published:** 2024-12-18

**Authors:** Lorenzo Giovannico, Giuseppe Fischetti, Domenico Parigino, Luca Savino, Nicola Di Bari, Aldo Domenico Milano, Massimo Padalino, Tomaso Bottio

**Affiliations:** Cardiac Surgery Unit, Department of Precision and Regenerative Medicine and Ionian Area (DiMePRe-J), University of Bari Aldo Moro, Bari, Italy

**Keywords:** heart transplant, heart failure, primary graft dysfunction, ECMO, heart disease

## Abstract

Heart failure is a serious and challenging medical condition characterized by the inability of the heart to pump blood effectively, leading to reduced blood flow to organs and tissues. Several underlying causes may be linked to this, including coronary artery disease, hypertension, or previous heart attacks. Therefore, it is a chronic condition that requires ongoing management and medical attention. HF affects >64 million individuals worldwide. Heart transplantation remains the gold standard of treatment for patients with end-stage cardiomyopathy. The recruitment of marginal donors may be considered an asset at the age of cardiac donor organ shortage. Primary graft dysfunction (PGD) is becoming increasingly common in the new era of heart transplantations. PGD is the most common cause of death within 30 days of cardiac transplantation. Mechanical Circulatory Support (MCS), particularly venoarterial extracorporeal membrane oxygenation (V-A ECMO), is the only effective treatment for severe PGD. VA-ECMO support ensures organ perfusion and provides the transplanted heart with adequate rest and recovery. In the new era of heart transplantation, early use allows for increased patient survival and careful management reduces complications.

## Introduction

On the night of 3 December 1967, Dr. Christiaan Barnard performed the first human heart transplant [1]. Since then, organ transplantation has made marked progress in reducing the morbidity and mortality of patients with heart failure (HF) [2]. Heart Transplantation remains the gold-standard therapy for end-stage HF. Although the number of transplants has increased over the decades, the greatest limitation in performing heart transplants is a lack of donors. In addition, patients who undergo heart transplantation are increasingly complex: elderly people with multiple pathologies have a higher level of calculated panel reactive antibody (cPRA) antigen sensitization (>20%), some of which undergo transplantation with short-term mechanical circulatory support (ST-MCS) [3]. Due of this situation, the use of marginal donors could be considered an asset provided there is a careful match between donor and recipient, and satisfactory results can be obtained both in the short and long term [4]. Several studies have aimed to expand selection criteria for marginal donors. As we find ourselves working with marginal donors and increasingly complex recipients, short-term mechanical circulatory support (ST-MCS) and venoarterial extracorporeal membrane oxygenation (V-A ECMO) will become increasingly fundamental. In 2018, in the USA, the United Network for Organ Sharing implemented a 6-tier allocation policy to replace the prior 3-tier system. The new allocation system allows stratification of candidates by mortality on the waiting list and reduces the time on the waiting list for patients with a higher priority. At the first evaluation performed by Maitra et al. in 2023, the change in the allocation system made it possible to improve the stratification of patients on the waiting list, to increase the number of patients arriving at the transplant with ST-MCS support (VA-ECMO or IABP), intubated, requiring hospitalization in intensive care, ischemia times and distance traveled for organ harvesting, and the use of marginal donors. At the same time, it reduced waiting list times, waiting list mortality, and post-heart transplant mortality in patients weaned from VA-ECMO support [5, 6].

This review aimed to analyze the use of venoarterial extracorporeal membrane oxygenation (ECMO) in the new era of cardiac transplantation.

## Recipient Characteristics

Those who have experienced the evolution of heart transplantation have noticed how over the years, the population of donors and recipients has changed significantly from 30 years ago.

Many centers worldwide have expanded acceptance criteria for adult heart transplant candidates. This, combined with changes in population demographics, has led to significant changes in the recipient characteristics [7].

Notably, the proportion of listed candidates aged>70 years increased from 2.5% in 2000 to 11% in 2017, reflecting the trend of an aging population and a greater focus on biological aging and other comorbidities. Guidelines regarding HT candidacy recommend that patients >70 years of age should be carefully selected, but do not provide further guidance [8–10].

Candidacy for HT depends on the intersection of chronological and biological age, and requires close investigation of social support, cognitive function, and frailty [8].

Allosensitized patients had elevated calculated panel reactive antibody (cPRA) values. Sensitizing events included pregnancies, blood transfusions, MCS devices, or prior surgeries [9].

Therefore, women and patients with LVADs are more likely to be sensitized. It can be difficult to find suitable donors with acceptable immunogenetic profiles for these patients, which can lead to longer waiting times and higher waitlist mortality [8].

Furthermore, there has been an increase in the number of recipients with diabetes, history of malignancy, history of pre-transplant dialysis, and previous cardiac surgery (Table 1). Although the number of hospitalized and mechanically ventilated patients and the use of inotropes have decreased, the number of patients in ST-MCS (ECMO) and LT-MCS (L-VAD) has increased (Table 2) [7].

**TABLE 1:** Adopted from [7].

	Jan 1992–Dec 2000 (N = 37,616)	Jan 2001–Dec 2009 (N = 33,588)	Jan 2010–Jun 2018 (N = 36,830)	P-value
PRA ≥ 20%	5.2%	10.0%	17.9%	<0.0001
PRA ≥ 80%	0.9%	2.3%	3.6%	<0.0001
Hep B antibody positive	3.8%^a^	5.2%	4.9%	<0.0001
Diabetes	16.7%^a^	22.3%	27.0%	<0.0001
History of malignancy	3.8%^a^	5.8%	8.7%	<0.0001
Pre-transplant dialysis	3.0%^a^	4.1%	4.8%	<0.0001
Previous cardiac surgery	37.8%	43.6%	50.1%	<0.0001

Summary statistics included transplants with known/non-missing data.

^a^ based on Apr 1994 to Dec 2000.

**TABLE 2:** Adopted from [7].

	Jan 1992–Dec 2000 (N = 37,616)	Jan 2001–Dec 2009 (N = 33,588)	Jan 2010–Jun 2018 (N = 36,830)	P-value
Inotrope use	46.7%^a^	43.4%	35.7%	<0.0001
IABP use	6.0%	6.2%	6.6%	0.0672
ECMO use	0.2%^b^	0.7%	1.1%	<0.0001
Type of MCS use				
- None	-	77.2%	55.6%	<0.0001
- VAD	-	18.9%	40.4%	
Ventilator use	3.4%	3.2%	1.7%	<0.0001
Hospitalized	60.7%	47.6%	44.6%	<0.0001

Summary statistics included transplants with known/non-missing data.

^a^ Based on Apr 1994 – Dec 2000 transplants.

^b^ Based on Apr 1995 – Dec 2000 transplants.

## Donor Characteristics

Considering the increase in HF patients worldwide and the changing recipient population, the use of marginal donors can be considered an additional resource after careful matching between donor and recipient to guarantee a heart transplant to those who need it and satisfy the heart transplant waiting list [11]. Several studies have aimed to expand selection criteria for marginal donors [12–15]. Controversial results have been reported on posttransplant outcomes following an MD donation, with relatively short-term data [13]. In Italy, according to the regulations of the National Transplant Center, MDs were defined according to the following criteria: age >60 years, reduced left ventricular performance (ejection fraction 40%–50%), left ventricular hypertrophy (septal thickness >14 mm on echocardiographic evaluation), focal lesion of the coronary artery, and significant valvular heart disease. The main concerns regarding the use of these donors are related to their increased susceptibility to high perioperative mortality, coronary graft vasculopathy, and primary graft failure (PGF) [13].

## Primary Graft Dysfunction

Primary graft dysfunction (PGD) is becoming increasingly common in the new era of heart transplantations. This syndrome is a dysfunction of the transplanted heart that occurs immediately following heart transplantation [16]. PGD is the most common cause of death within 30 days of cardiac transplantation [17], and the most recent report from the International Society for Heart and Lung Transplantation (ISHLT) Registry reported that 42.6% of deaths within 30 days of transplantation were due to PGD [18]. Several factors have been implicated in the development of PGD, including donor age, cause of donor cerebral death, organ preservation technique (e.g., cold storage), ischemic time >4 h, sex mismatch, preoperative presence of recipient VAD o ST-MCS, pulmonary hypertension, and elevated inotropic score [19–25]. Temporal, echocardiographic, hemodynamic and clinical criteria such as the use of high doses of inotropic drugs and/or mechanical support with ST-MCS are necessary to diagnose PGD.

(Table 3) The ISHLT recently published a consensus document with standardized criteria for PGD diagnosis following cardiac transplantation [26].

**TABLE 3:** Adopted from [25].

PGD left ventricle (PGD-LV):	Mild PGD-LV: one of the following criteria must be met:	LVEF <40% by echocardiography, or Haemodynamics with RAP >15 mmHg, PCWP >20 mmHg, CI < 2.0 L/min/m2 (lasting more than 1 h) requiring low-dose inotropes
	Moderate PGD-LV: must meet one criterion from I and another criterion from II:	I. LVEF <40%, or Hemodynamic compromise with RAP >15 mmHg, PCWP >20 mmHg, CI < 2.0 L/min/m2, hypotension with MAP <70 mmHg (lasting more than 1 h)
		II. High-dose inotropes—inotrope score >10a, or Newly placed IABP (regardless of inotropes)
	Severe PGD-LV	Dependence on left or biventricular mechanical support including ECMO LVAD, BiVAD, or percutaneous LVAD. Excludes requirement for IABP.
PGD right ventricle (PGD-RV):	Diagnosis requires either both i and ii, or iii alone:	i. Haemodynamics with RAP >15 mmHg, PCWP <15 mmHg, CI < 2.0 L/min/m2 ii. TPG <15 mmHg and/or pulmonary artery systolic pressure <50 mmHg iii. Need for RVAD

BiVAD, biventricular assist device; CI, cardiac index; ECMO, extracorporeal membrane oxygenation; IABP, intra-aortic balloon pump; MAP, mean arterial pressure; LVAD, left ventricular assist device; LVEF, left ventricular ejection fraction; PCWP, pulmonary capillary wedge pressure; PGD, primary graft dysfunction; RAP, right atrial pressure; RVAD, right ventricular assist device; TPG, trans pulmonary pressure gradient.

Inotrope score = dopamine (1) + dobutamine (1) + amrinone (1) + milrinone (15) + epinephrine (100) + norepinephrine (100) with each drug dosed in mg/kg/min.

Depending on the severity of the primary graft dysfunction, treatment may be based on the use of inotropic drugs or, in the most severe forms, on the use of short-term mechanical circulatory support.

MCS is the only effective treatment for severe PGD [21–25, 27–32]. ECMO support ensures organ perfusion, giving the transplanted heart time to rest and recover. The use of ECMO allows for a reduction in the support of inotropic drugs and vasoactive agents that stress the transplanted heart by increasing cardiac work, vasoconstriction, and afterload.

## V-A ECMO

Venoarterial extracorporeal membrane oxygenation guarantees immediate and complete support for patients with cardiogenic shock and cardiac arrest. The system consists of a centrifugal pump that can completely compensate for the cardiac output, a venous drainage cannula, and an arterial return cannula. A hollow fiber membrane oxygenator was positioned in the circuit to ensure oxygenation of venous blood and to remove carbon dioxide (CO_2_) via sweep gas flow.

## V-A ECMO Configurations

In our center, the sternum is closed after full reversal of heparin with protamine sulfate. Partial ECMO flow (2,2-3 L/min/m2) was maintained in conjunction with inotropes and inhaled nitric oxide, while the patient was intubated and sildenafil was administered when extubated to maintain ejection through the aortic and pulmonary valves. Anticoagulation with heparin (UFH) is initiated 24 h after sternal closure at a rate of 400 U/h. Activated clotting time (ACT) was measured every 4 h with a target of 180–200 s. Partial thromboplastin time was measured every 8 h, with a target of 45–60 s.

VA-ECMO can be performed surgically and percutaneously. When positioned surgically, it can be positioned with central cannulation (arterial cannula in the ascending aorta and venous drainage cannula in the right atrium) or peripherally via femoral or subclavian arterial access and the femoral vein.

The most commonly used access method at our center for patients with primary graft dysfunction is surgical or percutaneous femoral access. The venous cannula draining the deoxygenated venous blood was positioned in the superior vena cava-RA junction. After passing through the hollow fiber membrane oxygenator, oxygenated blood is returned to systemic circulation via an arterial cannula with its tip typically positioned in the iliac artery. Selecting cannulas with appropriate diameters is critical not only to reduce the risk of vascular injury, but also to avoid significant negative inflow (preferably< 50 mmHg) and high outflow pressure (<300 mmHg). At our center, to reduce the risk of distal limb ischemia, a 6Fr distal reperfusion cannula is routinely inserted into the superficial femoral artery [33, 34]. The use of peripheral and percutaneous access makes V-A ECMO support versatile and can be used in the operating room and bedside in the intensive care unit (ICU) [35, 36]. Peripheral cannulation allows for primary chest closure, diminishing the potential risks of chest infections and early respiratory weaning at the expense of a higher risk of limb ischemia, impaired unloading of the heart, and potential risk of harlequin syndrome [37].

Patients with central access had higher vasoactive-inotropic scores and more renal dysfunction without significant differences in lactate levels before MCS. Peripheral cannulation was associated with unrelated MCS infections and less MCS-related bleeding. Severe vascular complications were more frequent in patients with peripheral MCS, lymphorrhea at the access point, and venous thrombosis. No significant differences were observed in intubation time, stroke, embolism, or continuous renal replacement therapy (CRRT) [37].

## Unloading Left Ventricle

Recently, two large, randomized trials, EURO-SHOCK and ECLS-SHOCK, have been completed and published to address the question of whether VA-ECMO therapy in cardiogenic shock improves survival [38]. Our indications for venting the left ventricle included one or more of the following criteria: a high PCWP; Dilated, hypo-contractile LVX with blood pooling in the left ventricle seen on echocardiogram with increased LVEDP, aortic valve remains closed for the entire duration of the cardiac cycle, reduced oxygenation and persistent pulmonary edema shown on chest radiography, and ventricular arrhythmias.

In such cases, it is necessary to unload the left ventricle and place the heart “at rest’.” Therapeutic strategies for dealing with this complex situation are as follows:

• Inotropic drugs: These are the first-line intervention (dobutamine) to improve myocardial contractility and, therefore, left ventricular output.
• IABP: Opinions differ regarding the usefulness of IABP in this context. IABP causes a reduction in afterload due to the “vacuum” effect and some studies have suggested that it can improve coronary flow and therefore myocardial ischemia.
• reduction of congestion using diuretics and/or continuous renal replacement therapy (CRRT).
• Impella, a coaxial pump positioned retrogradely in the transvalvular aortic position, sucks blood from the left ventricle and expels it directly into the ascending aorta.
• left ventricular venting: this is the definitive treatment for left ventricular unloading.

The main venting methods are:

• Left atrial cannulation: Used especially in children, the drainage cannula is positioned directly in the left atrium through puncture of the atrial septum.
• Direct drainage of the left ventricle: Through surgical access, a drainage cannula is positioned at the apex of the left ventricle, which is connected in a Y-shape with the peripheral venous line (usually femoral), and then connected to the centrifugal pump and oxygenator.
• transaortic and axillary artery venting.
• Conversion of VA-ECMO to LVAD: A cannula is inserted into the left ventricle with an apical approach in a left anterior mini-thoracotomy to ensure optimal unloading of the left ventricle, and is then connected in a Y-shape with the venous cannula of the ECMO.

In a recent meta-analysis, Iannaccone et al. suggested that an ECpella vs. ECMO-only strategy for the management of CS may reduce 30-day mortality and increase LV recovery, despite increased bleeding rates and hemolysis [39].

A recent meta-analysis by Bathia et al. demonstrated that patients with cardiogenic shock requiring VA-ECMO support and concurrent LV unloading with Impella had a lower likelihood of short-term mortality and a higher likelihood of progression to durable LVAD or heart transplantation. However, patients supported with ECPELLA had higher rates of hemolysis, limb ischemia, and renal failure, requiring continuous renal replacement therapy [40].

Although the latter are the most effective venting systems, they are rarely used to unload the left ventricle during primary graft dysfunction after heart transplantation. Generally, an inotropic support and percutaneous device such as an IABP or Impella are more than sufficient for unloading the left ventricle.

## Ventilation on V -A ECMO

The goal of mechanical ventilation in these patients is to maintain adequate gas exchange while minimizing the risk of lung damage by placing the lungs at rest.

To keep the lung at rest and avoid the risk of volotrauma and barotrauma, it is recommended to maintain “protective ventilation,” setting a respiratory rate between 10 and 15 breaths/min, a tidal volume (tidal volume, Vt) ≤8 mL/kg and a plateau pressure ≤25 cmH2O, tolerating a certain degree of “permissive” hypercapnia.

In patients with severe cardiac impairment such as those with ECLS, high levels of positive end-expiratory pressure (PEEP) inhibit venous return and may have negative effects on hemodynamics. The PEEP values are usually set between 5 and 8 cmH2O.

Hyperoxia appears to be associated with increased mortality in patients with ECLS, as it causes a pro-inflammatory response to reactive oxygen species and induces vasoconstriction with consequent organ hypoperfusion [38]. Therefore, it is recommended to reduce the inspired fraction of oxygen (FiO2) to the lowest possible level (FiO2 0.40−0.60) with a target arterial oxygen pressure between 60 and 100 mmHg. In recent years, given the emergence of eco-support as a therapy for primary graft dysfunction, we have considered ways to reduce the complications that can occur when using this support. Many of these factors are associated with mechanical ventilation [39, 40].

Awake emergency strategies have been proposed to minimize complications related to mechanical ventilation. The findings described in case reports, pediatric cohorts, and a few series [41–44] indicate a better CS prognosis, but these studies were underpowered by the small number of patients enrolled and the inclusion of non-arousable patients with likely neurological impairment. Managing awake patients with V-A ECMO support is a relatively new and challenging task in the intensive care unit (ICU) [45].

The pros are:

• *Rehabilitation:* Muscle mass loss, critical illness, myopathy, and polyneuropathy often affect patients [46, 47].
• *Reduction of delirium:* The pathogenesis of delirium in the ICU is multifactorial; however, the use of sedative drugs is one of the main causes.
• *Interaction with relatives/friends and medical stuff:* The ability of an awake patient to communicate with friends and relatives can render an unfriendly ICU environment a more usual and easy-to-cope situation for both patients and visitors. Furthermore, it is possible for awake patients to communicate their symptoms and need medical staff [45].

The disadvantages include the risk factors for invasive device displacement, patient discomfort, pain, and anxiety. An awake patient required analgesics for invasive device tolerance and pain control [45]. Montero et al. analyzed 231 patients who underwent peripheral ECMO implantation. Of these 231 patients, 91 were “awake” and 140 were “not awake” After PS-matching adjustment, the “awake ECMO” group had significantly lower rates of pneumonia (35% vs. 59%, P = 0.017), tracheostomy, renal replacement therapy, and less antibiotic and sedative consumption. This strategy was also associated with reduced 60-day (20% vs. 41%, P = 0.018) and 1-year mortality rates (31% vs. 54%, P = 0.021) compared to the “non-awake” group, respectively [48]. In this paper, an “awake ECMO” is feasible, safe, and associated with improved short- and log-term outcomes.

## Weaning of V -A ECMO

Attempts to wean patients from ECMO were initiated after the first 48–72 h of ECMO assistance. ECMO support should be provided early in heart transplant patients with primary graft dysfunction to avoid organ damage. Therefore, because there are no organs damaged by low perfusion that need to be recovered, the indication for ECMO-weaning is generally indicated based on echocardiography, hemodynamics, and biological parameters.

The weaning criteria included MAP> 60 mmHg, PCWP< 18 mmHg, CI>2,4 L/min/m^2^; SVcO2 value ≥70%, hematocrit of 28%–30%; no bleeding or tamponade, left ventricular ejection fraction ≥25%, absence of dilation of the left ventricle, good contraction of the right ventricle (ejection fraction >25%) with absence of moderate or severe tricuspid regurgitation, normal blood lactate levels (<2 mmol/L), and normal diuresis (1-2 mL/kg/h) or dialysis.

Weaning should be initiated when patients are hemodynamically stable with a mean arterial pressure (MAP) > 60 mmHg in the absence or presence of low doses of inotropes and vasopressors for at least 24 h.

Weaning was gradual with reduction of the ECMO flow in steps of approximately 0.5–1.0 L/min/m^2^ (10% of the initial flow) approximately every 8-12 h with careful analyses of transesophageal or transthoracic echocardiography (Table 4) and evaluation of the values calculated by positioning the Swan-Ganz catheter (Table 5).

**TABLE 4:** Echocardiographic parameters to be evaluated in V-A ECMO weaning.

Echocardiographic monitoring
distention/overload of the left ventricle
Aortic velocity-time integral >10 cm
LVEF >25%
Lateral mitral annulus peak systolic > 6 cm/s
RVEF >25%
RV and LV strain
Recognition of complications
• Intracavitary thrombosis
• Cannula positioning/displacement
• Pericardial effusion/cardiac tamponade

**TABLE 5:** Hemodynamic parameters to be evaluated in V-A ECMO weaning.

Hemodynamic Parameters
MAP ≥60 mmHg
Pulmonary capillary Wedge pressure < 18 mmHg
Cardiac Index > 2,4 L/min/m^2^
Central Venous pressure < 18 mmHg
SvO2 e ScvO2
Lactate

ECMO flow is normally not reduced to below 1 L/min/m^2^ because of the high risk of circuit thrombosis at these flows. Once an ECMO flow of 1 L/min/m^2^ is reached, the pump flow is radically reduced to 0.5 L/min/m2 for approximately 15–30 min. If hemodynamics in terms of systemic arterial pressure (mean pressure >60 mmHg), left ventricular contractility (ejection fraction >25%), RVEF>25%, central venous pressure (10–15 mmHg), pulmonary capillary wedge pressure (10–15 mmHg), and SvO2 (>70%) are preserved, the chances of weaning will be very high. Inotrope support, if present, was maintained for at least 3 days after ECMO removal.

ECMO weaning tests are fundamental for evaluating the contractile function of the right ventricle, which is normally almost completely unloaded by a venous cannula positioned in the right atrium from the inferior vena cava during emergency support.

Few studies have aimed to identify the criteria for predicting which patients can be successfully weaned from ECMO using the same methodology. All of these studies considered clinical, hemodynamic, echocardiographic, or biological parameters. All these were observational studies, and the parameters were compared between weaned and non-weaned patients. The main limitations of these studies were the observational design and number of patients [49–54]. Ortuno et al. in a recent publication, Ortuno et al. investigated the best weaning strategy to use, and considering the data reported, proposed a strategy to optimize weaning from V-A ECMO.

## Anticoagulation

The Extracorporeal Life Support Organization (ELSO) guidelines for anticoagulation during ECMO recommend unfractionated heparin (UFH) as a 50–100 units/kg bolus at the time of cannulation, followed by a continuous infusion of 20–50 units/kg/h to achieve an activated clotting time (ACT) of 180–220 s [55]. Anticoagulation administered while using the V-A ECMO system should prevent the formation of clots, which could lead to embolisms or malfunction of the system, particularly of the oxygenator. In patients who have undergone a heart transplant, the use of V-A ECMO requires careful control and balancing of anticoagulation to prevent the formation of clots in the ecmo circuit and, at the same time, prevent bleeding. Desirable hemostatic balance is often difficult to achieve and may become a nearly impossible task in a high-bleeding-risk setting [56].

According to the 2021 ELSO Adult and Pediatric Anticoagulation Guidelines [57], monitoring anticoagulation using ACT is considered the gold standard for the guidance of anticoagulation therapy in patients with ECMO [58, 59]. In addition to the ACT, the APTT dosage is certainly fundamental and can help in assessing the patient’s level of anticoagulation. Furthermore, there is evidence that standard aPTT from the laboratory shows a stronger correlation with plasma UFH concentrations than ACT does [60].

The ELSO suggested repeating the test every 1-2 h in the case of ACT and every 6–12 h to monitor the anticoagulation effect with aPTT. [58, 59]. Some authors suggest the use of low molecular weight heparin (LMWH) and fondaparinux for anticoagulation in patients on VA ECMO. The primary method of monitoring both LMWH and fondaparinux is to measure anti-Xa levels [61, 62]. In our center, we use heparin (UFH) in Veno-Arterial ECMO. Anticoagulation with heparin (UFH) is initiated 24 h after sternal closure, and surgical bleeding was excluded at a rate of 400 U/h. Activated clotting time (ACT) was measured every 4 h, with a target of 180–200 s. Partial thromboplastin time was measured every 8 h, with a target of 45–60 s. In Table 6, we report the coagulation values based on various tests compared with the patient’s ECMO flow.

**TABLE 6:** Coagulation values based on the various tests compared to the patient’s ecmo flow.

	ACT	aPTT	Anti-Xa
Flow > 2,5 lt/min	160–180 s	35–40 s	0,15–0,20
Flow 2–2,5 lt/min	180–200 s	40–50 s	0,20–0,30
Flow < 2 lt/min	200–250 s	50–60 s	0,30

## Conclusion

Venoarterial extracorporeal membrane oxygenation (VA-ECMO) is a key player in controlling primary graft dysfunction (PGD) in patients undergoing heart transplantation. Through this strategy, organ perfusion of indispensable life support is guaranteed, and the transplanted heart is given sufficient time to regain normal function. The new age of heart transplantation has been marked by the earlier application of VA-ECMO, which decreases the risk of complications due to delayed mechanical support and increases survival rates.

Furthermore, the increasing use of marginal donors in the context of organ shortages and the increasing complexity of candidates waiting for transplantation make the use of high-performance mechanical support solutions indispensable for successful transplantation. VA-ECMO has a central role not only in PGD treatment but also in switching to either a long-term support system or a heart transplant, thus enhancing post-transplant outcomes.

The experience gained in heart transplantation and VA-ECMO support confirms the significance of enhanced ventilation and weaning techniques to minimize complications and improve short- and long-term prognoses. Systematic studies and meta-analyses will clarify the criteria for the optimal application of VA-ECMO and PGD management and provide a more stepped framework for healthcare professionals engaged in this delicate field.
